# Deep learning for lung cancer prognostication: A retrospective multi-cohort radiomics study

**DOI:** 10.1371/journal.pmed.1002711

**Published:** 2018-11-30

**Authors:** Ahmed Hosny, Chintan Parmar, Thibaud P. Coroller, Patrick Grossmann, Roman Zeleznik, Avnish Kumar, Johan Bussink, Robert J. Gillies, Raymond H. Mak, Hugo J. W. L. Aerts

**Affiliations:** 1 Department of Radiation Oncology, Dana-Farber Cancer Institute, Brigham and Women’s Hospital, Harvard Medical School, Boston, Massachusetts, United States of America; 2 Department of Radiation Oncology, Radboud University Medical Center, Nijmegen, The Netherlands; 3 Department of Cancer Physiology, H. Lee Moffitt Cancer Center and Research Institute, Tampa, Florida, United States of America; 4 Department of Radiology, Brigham and Women’s Hospital, Harvard Medical School, Boston, Massachusetts, United States of America; University of California San Francisco, UNITED STATES

## Abstract

**Background:**

Non-small-cell lung cancer (NSCLC) patients often demonstrate varying clinical courses and outcomes, even within the same tumor stage. This study explores deep learning applications in medical imaging allowing for the automated quantification of radiographic characteristics and potentially improving patient stratification.

**Methods and findings:**

We performed an integrative analysis on 7 independent datasets across 5 institutions totaling 1,194 NSCLC patients (age median = 68.3 years [range 32.5–93.3], survival median = 1.7 years [range 0.0–11.7]). Using external validation in computed tomography (CT) data, we identified prognostic signatures using a 3D convolutional neural network (CNN) for patients treated with radiotherapy (*n* = 771, age median = 68.0 years [range 32.5–93.3], survival median = 1.3 years [range 0.0–11.7]). We then employed a transfer learning approach to achieve the same for surgery patients (*n* = 391, age median = 69.1 years [range 37.2–88.0], survival median = 3.1 years [range 0.0–8.8]). We found that the CNN predictions were significantly associated with 2-year overall survival from the start of respective treatment for radiotherapy (area under the receiver operating characteristic curve [AUC] = 0.70 [95% CI 0.63–0.78], *p* < 0.001) and surgery (AUC = 0.71 [95% CI 0.60–0.82], *p* < 0.001) patients. The CNN was also able to significantly stratify patients into low and high mortality risk groups in both the radiotherapy (*p* < 0.001) and surgery (*p* = 0.03) datasets. Additionally, the CNN was found to significantly outperform random forest models built on clinical parameters—including age, sex, and tumor node metastasis stage—as well as demonstrate high robustness against test–retest (intraclass correlation coefficient = 0.91) and inter-reader (Spearman’s rank-order correlation = 0.88) variations. To gain a better understanding of the characteristics captured by the CNN, we identified regions with the most contribution towards predictions and highlighted the importance of tumor-surrounding tissue in patient stratification. We also present preliminary findings on the biological basis of the captured phenotypes as being linked to cell cycle and transcriptional processes. Limitations include the retrospective nature of this study as well as the opaque black box nature of deep learning networks.

**Conclusions:**

Our results provide evidence that deep learning networks may be used for mortality risk stratification based on standard-of-care CT images from NSCLC patients. This evidence motivates future research into better deciphering the clinical and biological basis of deep learning networks as well as validation in prospective data.

## Abstract 2

Hugo Aerts and colleagues evaluate the ability of deep learning networks to extract relevant features from computed tomography lung cancer images and stratify patients into low and high mortality risk groups.

## Author summary

**Why was this study done?:**

- Cancer is one of the leading causes of death worldwide, with lung cancer being the second most commonly diagnosed cancer in both men and women in the US.
- Prognosis in lung cancer patients is primarily determined through tumor staging, which in turn is based on a relatively coarse and discrete stratification.
- Radiographic medical images offer patient- and tumor-specific information that could be used to complement clinical prognostic evaluation efforts.
- Recent advances in radiomics through applications of artificial intelligence, computer vision, and deep learning allow for the extraction and mining of numerous quantitative features from radiographic images.

**What did the researchers do and find?:**

- We designed an analysis setup comprising 7 independent datasets across 5 institutions totaling 1,194 patients with non-small-cell lung cancer imaged with computed tomography and treated with either radiotherapy or surgery.
- We evaluated the prognostic signatures of quantitative imaging features extracted through deep learning networks, and assessed their ability to stratify patients into low and high mortality risk groups as per a 2-year overall survival cutoff.
- In patients treated with surgery, deep learning networks significantly outperformed models based on predefined tumor features as well as tumor volume and maximum diameter.
- In addition to highlighting image regions with prognostic influence, we evaluated the deep learning features for robustness against physiological imaging artifacts and input variability, as well as correlated them with molecular information through gene expression data.

**What do these findings mean?:**

- We found that deep learning features significantly outperform existing prognostication methods in surgery patients, hinting at their utility in patient stratification and potentially sparing low mortality risk groups from adjuvant chemotherapy.
- We demonstrated that areas within and beyond the tumor—especially the tumor–stroma interfaces—had the largest contributions to the prognostic signature, highlighting the importance of tumor-surrounding tissue in patient stratification.
- Preliminary genomic associations in this study suggest correlations between the deep learning feature representations and cell cycle and transcriptional processes.
- Despite their obscure inner workings and lack of a strong theoretical backing, deep learning networks demonstrate a prognostic signal and robustness against specific noise artifacts. This finding motivates further prospective studies validating their utility in patient stratification and the development of personalized cancer treatment plans.

## Introduction

Cancer’s ever-evolving nature and interaction with its surroundings continue to challenge patients, clinicians, and researchers alike. One of its deadliest forms appears in the lungs, leading to the most cancer-related mortalities worldwide [1]. Lung cancer is the second most commonly diagnosed cancer in both men and women [2], with non-small-cell lung cancer (NSCLC) comprising 85% of cases [3]. The ability to accurately categorize NSCLC patients into groups structured around clinical factors represents a crucial step in cancer care. This stratification allows for evaluating tumor progression, establishing prognosis, providing standard terminologies for effective clinical communication, and, most importantly, identifying appropriate treatment plans from chemotherapy and surgery to radiation and targeted therapy. In addition to clinical factors (including performance status) and, to a lesser extent, age and sex [4], tumor stage—as evaluated through the predominant tumor node metastasis (TNM) staging manual [5]—is often regarded as a universal benchmark for performing such stratification.

The predominant TNM staging manual represents a body of knowledge combining evidence-based findings from clinical studies with empirical knowledge from site-specific experts [6]. However, we find that patients within the same stage can exhibit wide variations in their response to treatment [7]. This owes, in part, to the inevitable gap that exists between yesterday’s statistics and today’s more advanced treatment options, as well as the practical challenges of stratifying patients into groups that fit historical data while balancing the ability of clinicians to identify the stratification features and apply the stratification algorithm at the point of care [8]. The limitations of our clinical gold standards, combined with our improved understanding of intra-tumor heterogeneity [9], signal the need for developing personalized biomarkers that can operate at the individual patient level—as opposed to the population level—eventually leading to more robust patient stratification and building a foundation for precision oncology practices.

The aforementioned clinician-driven stratification algorithms used in NSCLC staging rely on high-level semantic features describing tumor extent, location, and metastatic status. These are often inferred from standard medical images of the upper abdomen and thorax. These non-invasive images, however, offer information that goes beyond that captured through routine radiographic evaluation. Hardware advances in high-resolution image acquisition equipment and computational processing power, coupled with novel artificial intelligence (AI) algorithms and large amounts of data, have contributed to a proliferation of AI applications in radiology, medicine, and beyond. These have enabled the high-throughput extraction, and subsequent processing, of high-dimensional quantitative features from images. More specifically, this dialogue between AI and medical imaging has been recently manifested in radiomics.

Radiomics is a data-centric field involving the extraction and mining of quantitative features as a means to quantify the solid tumor radiographic phenotype [10]. It hypothesizes that radiographic phenotypes represent underlying pathophysiologies and are thus capable of discriminating between disease forms as well as predicting prognosis and therapeutic response [11]. Radiomics research has primarily relied on explicitly programmed algorithms that extract engineered (hand-crafted) imaging features. Such features commonly represent tumor shape, voxel intensity information (statistics), and patterns (textures). More specifically within oncology, radiomics has demonstrated success in stratifying tumor histology [12], tumor grades [13], and clinical outcomes [10]. Additionally, associations with underlying gene expression patterns have also been reported [14]. Given these associations, radiomic features have been used to build prognostic and predictive models making use of statistical machine learning algorithms coupled with feature selection strategies [15]. More recent work, however, has shifted towards deep learning as the de facto machine learning approach [16].

Deep learning has shown great promise in areas that rely on imaging data, including radiology [17], pathology [18], dermatology [19], and ophthalmology [20] to name a few. In lieu of the often subjective visual assessment of images by trained clinicians, deep learning automatically identifies complex patterns in data and hence provides evaluations in a quantitative manner. In contrast with feature engineering approaches, deep learning networks allow for the automated quantification and selection of the most robust features, and thus they require little to no human input. Deep learning methods have outperformed their engineered feature counterparts in many tasks including mammographic lesion detection [21], mortality prediction [22], and multimodal image registration [23].

Convolutional neural networks (CNNs) are a class of deep learning models that combine imaging filters with artificial neural networks through a series of successive linear and nonlinear layers. CNN layers learn increasingly higher level features from images, eventually making predictions by essentially mapping image inputs to desired outputs. CNNs have demonstrated great potential in various classification [24], detection [25], segmentation [26], registration [27], and reconstruction [28] tasks—learning from photographic, pathology, and radiographic images [17]. Other efforts use pretrained networks on images from other domains, an approach known as transfer learning [29], as a workaround when sample size is perceived to be insufficient. In some instances, classifiers are built using a combination of deep learning and engineered features [30]. However, and with a few exceptions, most studies lack generalization power due to insufficient data—usually under 100 patients. With such limited data, and to avoid overfitting, most efforts have been confined to solving 2D problems, or, alternatively, a 3D problem space is often treated as a composition of 2D orthogonal planes [31], with a few recent studies capitalizing on information within the entire 3D tumor volume [32]. To our knowledge, no studies to date have explored medical-to-medical transfer learning, with learned representations usually being transferred from general imagery. Only a few studies have assessed the stability of deep learning features extracted from medical images, with most solely relying on the presumed robustness of CNNs in other application areas.

**Fig 1:**
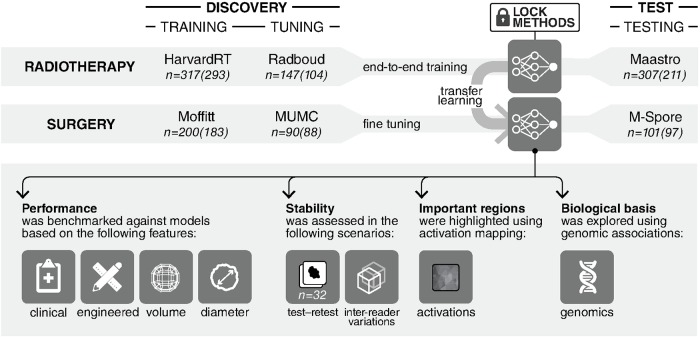
General design of the analytical setup. 3D convolutional neural network is trained end-to-end on the radiotherapy dataset group. This is followed by a transfer learning approach, where the same network is fine-tuned on the surgery dataset group. The training, tuning, and testing of these networks are all carried out on independent datasets as illustrated. Four further experiments are carried out on the networks in order to benchmark their performance against random forest models, assess their stability, identify regions in images responsible for predictions, and finally, explore their biological basis. Numbers outside parentheses refer to the number of patients with survival follow-up per dataset. Numbers within parentheses refer to the number of patients with 2-year overall survival follow-up only. Refer to Methods for patient censoring information and S1 Table for further dataset breakdown and information.

In this study, we investigated the ability of deep learning networks, 3D CNNs in particular, to quantify radiographic tumor characteristics and predict overall survival likelihood. We designed a rigorous analytical setup (Fig 1), with 7 large and independent datasets of 1,194 NSCLC patients imaged with computed tomography (CT) across 5 institutions, to discover and validate the prognostic power of CNNs in patients treated with radiotherapy and surgery. The prognosis is formulated as a binary 2-year overall survival classification problem. We benchmarked the CNN’s performance against models built on clinical parameters and engineered features, as well as demonstrated its stability in both test–retest and inter-reader variability scenarios. To gain a better understanding of the characteristics captured by the CNN, we mapped salient regions in images as per their contributions to predictions, both within and beyond the tumor. Additionally, we aimed at assessing the driving biological pathways as a means to explore the biological basis of the captured phenotypes. Our results highlight the improved performance of deep learning networks over their engineered counterparts, their robustness against specific types of input variability, their perceived biological basis, and their ultimate potential in improving patient stratification.

## Methods

### Datasets

We utilized 7 independent datasets in this study (S1 Table; S1 Text)—3 radiotherapy datasets, 3 surgery datasets, and a stability assessment dataset. They come from a combination of European and US institutions as well as open-access online repositories.

#### Radiotherapy dataset group

The radiotherapy dataset group consists of the following datasets:

- **HarvardRT** (training) consists of 317 NSCLC stage I–IIIb patients imaged with CT, with or without intravenous contrast, and treated with radiation therapy at the Dana-Farber Cancer Institute and Brigham and Women’s Hospital, Boston, Massachusetts, US. Images were acquired between 2001 and 2015.
- **Radboud** (tuning) consists of 147 NSCLC stage I–IIIb patients imaged with contrast-enhanced CT and treated with radiation therapy at Radboud University Medical Center, Nijmegen, The Netherlands. Images were acquired between February 2004 and October 2011.
- **Maastro** (testing) consists of 307 NSCLC stage I–IIIb patients, imaged with CT, with or without intravenous contrast, and treated with radiation therapy at MAASTRO Clinic, Maastricht, The Netherlands. Images were acquired between 2004 and 2010. This dataset is available at https://wiki.cancerimagingarchive.net/display/Public/NSCLC-Radiomics.

#### Surgical dataset group

The surgical dataset group consists of the following datasets:

- **Moffitt** (training) consists of 200 NSCLC stage I–IIIb patients imaged primarily (89%) with contrast-enhanced CT and treated with surgical dissection at the Thoracic Oncology Program at the H. Lee Moffitt Cancer Center, Tampa, Florida, US. Images were acquired between 2006 and 2009.
- **MUMC** (tuning) consists of 90 NSCLC stage I–IIIb patients, imaged with CT, with or without intravenous contrast, and treated with surgical dissection at MAASTRO Clinic, Maastricht, The Netherlands. Images were acquired between 2004 and 2010. This dataset is available at https://wiki.cancerimagingarchive.net/display/Public/NSCLC-Radiomics-Genomics.
- **M-SPORE** (testing) consists of 101 NSCLC stage I–IIIb patients imaged with contrast-enhanced CT and treated with surgical dissection at the Thoracic Oncology Program at the H. Lee Moffitt Cancer Center, Tampa, Florida, US. Images were acquired between 2006 and 2009.

#### Stability assessment dataset

The stability assessment dataset comprises the following:

- **RIDER** consists of 32 patients with NSCLC, each of whom underwent 2 CT scans of the chest within 15 minutes [33]. Images were acquired between January 2007 and September 2007. This dataset is available at https://wiki.cancerimagingarchive.net/display/Public/RIDER+Collections.

Overall survival times were calculated from the start of respective treatment for the radiotherapy and surgery datasets. These continuous survival times were dichotomized using a 2-year cutoff. Datasets were then right-censored; alive patients at a last known follow-up of less than 2 years were excluded. This setup allows for a binary 2-year survival endpoint of 0 for deceased patients and 1 for alive patients—relative to the 2-year cutoff. To ensure non-biased dataset assignment for training, tuning, and testing, datasets with the most and least patients were assigned as training and tuning, respectively. The remaining dataset was locked for testing. This assignment system was applied to both the radiotherapy and surgery dataset groups. Initial experiments were done on the radiotherapy datasets as they contained the most data, followed by transfer learning and fine-tuning on the surgery datasets. This design also allowed for averting noise as a result of large variability in tumor sizes between the 2 dataset groups, with the surgery group comprising consistently smaller tumors on average. All patients were utilized as per the survival data available, without introducing artificial temporal cutoffs.

### Data preprocessing

Tumors were manually contoured and approved by an expert reader (S1 Text). With slice thickness exceeding in-plane resolution, all datasets were resampled into isotropic voxels of unit dimension to ensure comparability, where 1 voxel corresponds to 1 mm^3^. This was achieved using linear and nearest neighbor interpolations for the image and annotations, respectively. If multiple disconnected annotation masks were found, the largest by volume was chosen.

### Data preprocessing for deep learning

Given full 3D tumor segmentations, both the center of mass (COM) and bounding box of the tumor annotations were calculated. 3D isotropic patches of size 50 × 50 × 50 were extracted around each COM, capturing around 60% of the tumor bounding boxes’ dimensions in the radiotherapy training dataset (S1 Fig). The patches were then normalized to a 0–1 range using lower and upper Hounsfield unit bounds of −1,024 and 3,071, respectively. An augmentation factor of 32,000 was applied to the patches, yielding a training size of approximately 9.4 million and 5.9 million input samples for the radiotherapy and surgery datasets, respectively. These augmentations included random translations ±10 pixels in all 3 axes, random rotation at 90° intervals along the longitudinal axes only, and random flipping along all 3 axes. Augmentation was done in real time during training. No tuning- or testing-time augmentation was applied.

### Deep learning

**Fig 2:**
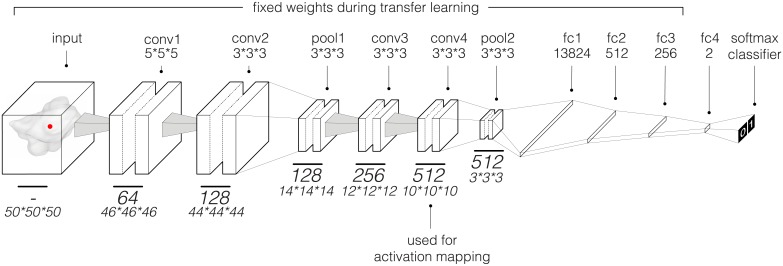
Illustration of the convolutional neural network. This network was used to predict overall 2-year survival of patients with non-small-cell lung cancer. The final classifier layer outputs normalized probabilities for both classes (0 = deceased and 1 = alive). Only the weights of the final fully connected layer were fine-tuned during transfer learning. The final convolutional layer (conv4) was used for activation mapping.

We employed a 3D CNN architecture (Fig 2). The network comprises a total of 4 3D convolutional layers of 64, 128, 256, and 512 filters with kernel sizes of 5 × 5 × 5, 3 × 3 × 3, 3 × 3 × 3, and 3 × 3 × 3, respectively. Two max pooling layers of kernel size 3 × 3 × 3 were applied after the second and fourth convolutional layers. A series of 4 fully connected layers—with 13,824, 512, 256, and 2 units—provided high-level reasoning before the prediction probabilities were calculated in the final softmax classifier layer. Training details are as follows: We used the gradient-based stochastic optimizer Adam [34] with a global learning rate of 1 × 10^−03^ without decay, a batch size of 16, dropout [35] of 25% and 50% on the convolutional and fully connected layers, respectively, and a L2 regularization [36] penalty term of 1 × 10^−05^. To avoid the internal covariance shift problem [37], batch normalization was applied across all layers, with the input layer as an exception. Leaky rectified linear units (leaky ReLUs) [38] with alpha = 0.1 were the activation function of choice across the entire network prior to the final softmax activation. In training the CNN within the radiotherapy dataset, we used a random grid search exploring different hyper-parameters including input patch size, batch size, learning rate, regularization term, and convolution kernel size. As for the general architecture, we started with a shallow network, where underfitting occurs, and incrementally added layers. The model was optimized on the tuning dataset using early stopping [39]. With a 1,000-epoch limit, the model with the best performance on the tuning dataset was chosen. In applying transfer learning on the surgery training dataset, the number of final layers to fine-tune was explored. The optimal setting included fine-tuning the final classification layer only, while keeping earlier layers fixed. With much fewer parameters to train, the learning rate and batch size were increased to 1 × 10^−02^ and 24, respectively. Google’s deep learning framework TensorFlow [40] was used to train, tune, and test the CNN.

### Data preprocessing for engineered feature extraction

Image intensity was binned by 25 HU to increase pattern sensitivity. Preprocessing filters were applied prior to feature extraction in order to reveal underlying information. These included Laplacian of Gaussian, wavelet, square, exponential, square root, and logarithm filters.

### Engineered feature extraction and selection

Engineered features were computed using PyRadiomics [41], an open-source radiomics package. Feature stability was quantified using the intraclass correlation coefficient (ICC), using the irr package [42] and the test–retest RIDER dataset [33,43]. Features with an ICC > 0.8 were regarded as highly robust and selected for the study. Supervised selection was done using the mRMR method (minimum redundancy maximum relevance) with the mRMRe package [44]. The mRMR was applied on the tuning datasets to select the top 40 engineered features with the highest mRMR ranks. Those features were then used for the final model on the training and testing datasets.

### Machine learning on clinical parameters and engineered features

A random forest classifier was built using clinical parameters and engineered features. The tuning process involved a nested cross-validation technique (5,000-fold, 5 times) using the caret package [45] on the training dataset to select the best parameters, such as the number of variables randomly sampled. The predictive power was measured on the testing dataset using the area under the receiver operating characteristic curve (AUC). Significant difference from random permutation was tested using a 2-sided Wilcoxon rank-sum test between the score of the 2 classes.

### Benchmarking

Benchmarking of deep learning networks against other models was done using a permutation test. AUC difference was defined as a Δ. For *N* permutations (*N* = 1,000 in our case), new models were built after randomly permuting class labels, and new AUCs were computed from their respective scores. The new difference Δ*i* was then converted to 0 if below Δ or 1 if above. Finally, the *p*-value was defined as

$$p=\frac{1}{N}\sum_{i}^{N}\Delta i;\;where\;\Delta i=0\;if\;\Delta i<\Delta ,\Delta i=1\;if\;\Delta i>\Delta$$

If the AUC difference between those 2 random models was higher than the true value, then the true class label was randomly permuted. A new model was then built, and its score distribution was compared to the true distribution. Finally, a meta *p*-value was computed combining the results of the radiotherapy and surgery datasets using the survcomp package [46].

### Activation mapping

To generate activation maps, we used a gradient-weighted activation mapping method [47,48] to map important regions in an input image with respect to predictions made. The final convolutional layer (conv4 in Fig 2) was set as the penultimate layer where the activation heatmaps (gradients) were generated during backpropagation. The heat maps were then thresholded at 0, normalized, and enlarged to match the input image size. The heatmaps indicate regions in the input image having the most impact on the final prediction layer.

### Masking experiment

Ground truth tumor annotations were used to delineate tumor areas, and all voxels beyond the annotations were given the value of air (−1,000 HU). The deep learning network was retrained with the masked data while keeping all hyper-parameters locked.

### Genomic studies

We performed a pre-ranked gene set enrichment analysis (GSEA) as in previously published studies [14,49,50]. Briefly, more than 60,000 probes measured global gene expression on custom Affymetrix 2.0 microarray chipsets (HuRSTA_2a520709.CDF, GEO accession number GPL15048). Measured expression was normalized according to the robust multi-array average method [51]. Expression values were correlated with the network predictions to create a rank of all genes using Spearman rank correlation coefficients. This gene rank was input to a pre-ranked version of GSEA [52]. GSEA calculates scores that quantify the association of a given rank of genes with a predefined list of gene sets representing biological pathways. In such manner, GSEA allows for understanding what biological types of pathways the rank of genes corresponds to. As gene sets, we tested expert-curated pathways from the C2 Reactome collection version 6 available at MSigDB [53] using the GSEA software version 3 with 1,000 permutations. Gene sets were restricted to sizes between 5 and 500, resulting in 669 tested gene sets. Expression data are publically available via [14] and at https://www.ncbi.nlm.nih.gov/geo/query/acc.cgi?acc=GSE58661. We used the GSEA software’s Normalized Enrichment Score (NES) to quantify the association of the rank of genes with pathways and validated the NES with the false discovery rate (FDR) as per [54] to correct for multiple hypothesis testing.

## Results

### Tumor characterization using 3D deep learning networks

In assessing the ability of deep learning networks to quantify radiographic characteristics of tumors, we performed an integrative analysis on 7 independent datasets totaling 1,194 patients (Fig 1; S1 Table). We identified and independently validated prognostic signatures using a CNN for patients treated with radiotherapy (*n* = 771, including 608 with 2-year survival follow-up). We then employed a transfer learning approach to achieve the same for surgery patients (*n* = 391, including 368 with 2-year survival follow-up). The architecture of the network (Fig 2) was designed to receive 3D input cubes surrounding the center of the primary tumor—based on clinician-located seed points. The network was trained to predict overall survival likelihood 2 years after the start of the respective treatment.

**Fig 3:**
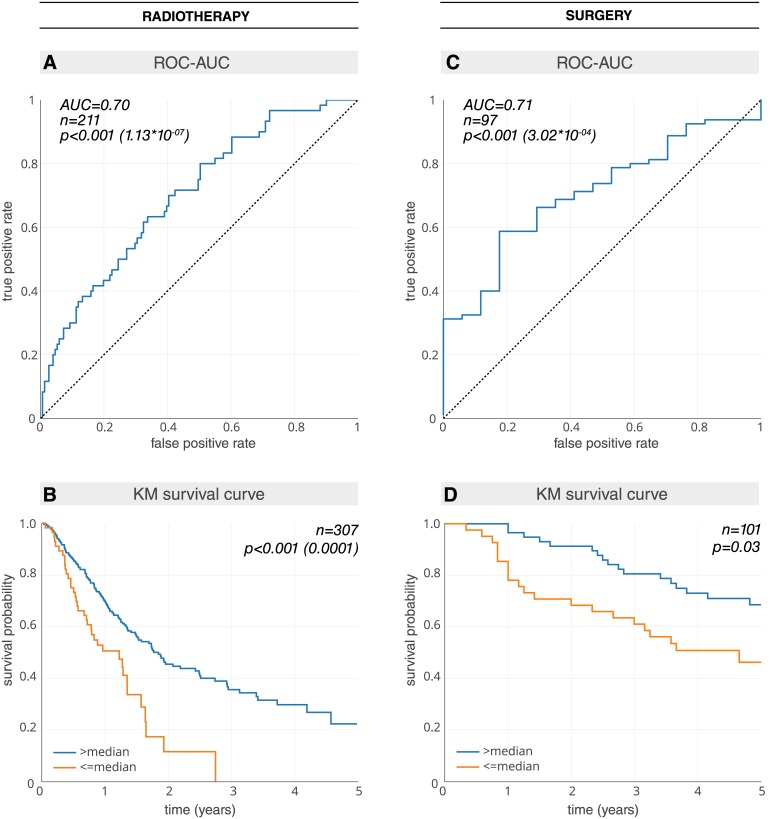
Prognostic power (AUC) and Kaplan–Meier (KM) curves of deep learning features for both the radiotherapy and surgical networks. (A) AUC plot for the radiotherapy test dataset Maastro (*n* = 211). (B) KM plot for the Maastro dataset (*n* = 307). Patients who have been previously excluded for lack of 2-year survival follow-up have been reincluded (S1 Table). To ensure an independent evaluation, the median split is calculated on the radiotherapy tuning dataset Radboud (*n* = 147) and locked for evaluation on the radiotherapy test dataset Maastro. (C) AUC plot for the surgery test dataset M-SPORE (*n* = 97). (D) KM plot for the M-SPORE dataset (*n* = 101). The median split is calculated on the surgery tuning dataset MUMC (*n* = 90) and locked for evaluation on the surgery test dataset M-SPORE. AUC or ROC-AUC, area under the receiver operating characteristic curve.

Starting with the radiotherapy patients, the analysis was split into a discovery phase and an independent test phase (Fig 1; S1 Table). Within the discovery phase, a 3D CNN was trained on the HarvardRT dataset (age median = 69.6 years [range 32.5–93.3], male/female = 140/153, survival median = 2.2 years [range 0.0–11.7], 2-year survival deceased/alive = 134/159) using augmentation, while the independent Radboud dataset (age median = 65.9 years [range 44.4–85.9], male/female = not available, survival median = 0.9 years [range 0.1–8.2], 2-year survival deceased/alive = 76/28) was used to iteratively tune and optimize the CNN’s hyper-parameters as well as the tumor 3D input patch sizes (S1 Fig; Methods) until the best prediction score was achieved. Beyond this discovery phase, the prognostic CNN was locked and tested on the independent Maastro dataset (age median = 69.0 years [range 34.0–91.7], male/female = 142/69, survival median = 1.0 years [range 0.0–5.8], 2-year survival deceased/alive = 151/60). The CNN showed a significant prognostic power in predicting 2-year survival (AUC = 0.70 [95% CI 0.63–0.78], *p* < 0.001) (Fig 3A). Kaplan–Meier curve analysis was performed to evaluate the CNN’s performance in stratifying low and high mortality risk groups. A significant survival difference (*p* < 0.001) was observed between the 2 groups on the independent Maastro dataset (Fig 3B).

In order to develop a prognostic deep learning network for surgical patients, we employed a transfer learning approach (Fig 1; S1 Table). The final prediction layers of the radiotherapy-trained CNN were fine-tuned on the Moffitt dataset (age median = not available, male/female = 83/100, survival median = 2.8 years [range 0.0–6.3], 2-year survival deceased/alive = 50/133) using augmentation (Fig 2; Methods). The independent MUMC dataset (age median = 68.0 years [range 37.2–83.3], male/female = 61/27, survival median = 3.3 years [range 0.2–8.8], 2-year survival deceased/alive = 24/64) was used to iteratively tune and optimize the CNN’s hyper-parameters as well as identify the optimum layers for fine-tuning. The CNN was then locked and tested on the independent test dataset M-SPORE (age median = 70.0 years [range 46.0–88.0], male/female = 44/53, survival median = 4.5 years [range 0.3–7.8], 2-year survival deceased/alive = 17/80), where it demonstrated a significant prognostic performance (AUC = 0.71 [95% CI 0.60–0.82], *p* < 0.001) (Fig 3C). Kaplan–Meier curve analysis showed a significant survival difference (*p* = 0.03) between low and high mortality risk groups within the M-SPORE test dataset (Fig 3D).

### Benchmarking against clinical parameters and engineered imaging features

The deep learning networks were benchmarked against random forest models based on clinical information (age, sex, and TNM stage). These clinical models achieved a performance of AUC = 0.55 (95% CI 0.47–0.64, *p* = 0.21) and AUC = 0.58 (95% CI 0.39–0.77, *p* = 0.4) for the radiotherapy and surgery datasets, respectively. Additionally, univariate analysis suggested that these clinical variables did not have a significant association with survival (S2 Table). Deep learning performed significantly better for both treatment types (S2 Fig).

The deep learning networks were also compared to random forest models based on engineered features describing tumor shape, voxel intensity information (statistics), and patterns (textures). The engineered feature models demonstrated a prognostic performance of AUC = 0.66 (95% CI 0.58–0.75, *p* < 0.001) and AUC = 0.58 (95% CI 0.44–0.75, *p* = 0.275) for the radiotherapy and surgery datasets, respectively (S2 Fig). Although the deep learning networks demonstrated improved performance over the engineered models for both patient groups, this difference was not significant for radiotherapy patients (*p* = 0.132; permutation test, *N* = 1,000), but was significant for surgery patients (*p* = 0.035; permutation test, *N* = 1,000). These results were confirmed with a meta *p*-value test (*p* = 0.06).

Finally, the deep learning networks were compared to imaging parameters commonly used in clinical practice, namely tumor volume and maximum diameter. We found that tumor volume achieved a performance of AUC = 0.64 (95% CI 0.56–0.73, *p* < 0.001) and AUC = 0.51 (95% CI 0.37–0.66, *p* = 0.85) for the radiotherapy and surgery datasets, respectively. The deep learning networks were borderline non-significantly better on the radiotherapy dataset (*p* = 0.056), and significantly better for the surgery dataset (*p* = 0.004), as confirmed with a meta *p*-value test (*p* <0.001). Similar results were found for maximum diameter (S2 Fig).

### Stability of deep learning networks

To evaluate the stability of the deep learning networks, we tested robustness against test–retest scenarios as well as inter-reader variations in input seed annotations. We used the publicly available test–retest RIDER dataset comprising 32 patients with lung cancer, each of whom underwent 2 chest CT scans within 15 minutes using the same imaging protocol and in a similar position [33]. Using this dataset, we evaluated the stability of network predictions between the test and retest scans. A high stability was demonstrated (ICC = 0.91) between both predictions.

To assess stability against inter-reader variations in input data, we randomly relocated the input seed points in 3D space around the center of the tumor (S3 Fig). This randomly shifts the network inputs during testing and can be regarded as simulating multiple human readers annotating the tumor’s center, with the inevitable variability among them. The network outputs show high correlation (Spearman’s rank-order correlation = 0.88). We also observed a high stability in prognostic predictions (AUC, μ = 0.68, σ = 0.014) (S3 Fig).

### Activation mapping of deep learning networks

**Fig 4:**
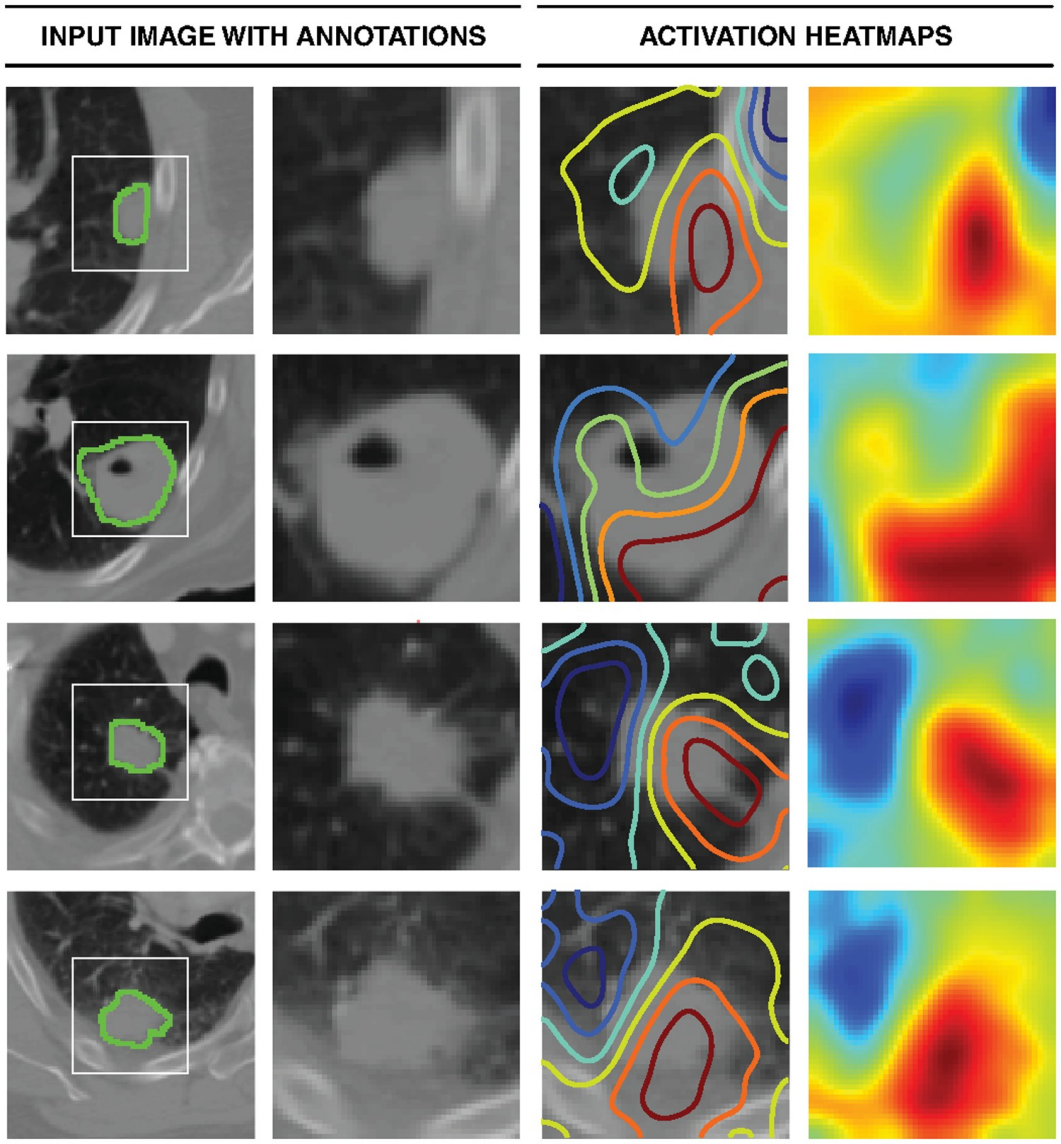
Activation mapping. Visual highlights of the most “important” regions within the input image—those with the most contribution to maximizing the outputs of the final prediction layer. The rows represent 4 randomly selected samples. From the left, the first column represents the central axial slice of the network input (150 × 150 mm) with tumor annotations. In the second column, a 50 × 50 mm patch is cropped around the tumor. In the third column, activation contours are overlaid, with blue and red showing the lowest and highest contributions (gradients), respectively. Column 4 represents the activation heatmaps for a better visual reference. While the heatmaps are 3D, only the central axial slice is shown. Therefore, the entire color spectrum might not be fully visualized.

To gain an understanding of regions within the CT images responsible for network predictions, we mapped the network’s activation maps over the final convolutional layer (Fig 4). The magnitudes of gradients flowing through this layer were used to decide on the “importance” of each node or voxel relative to the final prediction layer. This analysis allowed us to highlight the most relevant regions, with the most impact on predictions, both within and beyond the tumor. We observed that the network tended to fixate on the interface between the tumor and stroma (parenchyma or pleura). Most contributions to predictions came in the form of large uninterrupted areas of relatively higher CT density—spanning regions within and beyond the tumor. Areas with lower CT density, however, contributed the least to predictions. Examples of these include lobe areas with infrequent vessels or jagged interfaces between low and high CT density areas. We also observed that normal tissue, such as high-density bone tissue, was disregarded—as it is likely to exist in most images and is thus non-informative. This visual mapping demonstrates that tissue within and beyond the tumor were both crucial for characterization and eventual prediction. In order to further validate these findings, we retrained the deep learning network with masked images—essentially discarding data beyond the tumor. A drop in prognostic power was observed (from AUC = 0.70 to 0.63) (S4 Fig), hinting at the existence of discriminative texture features in tumor-surrounding regions.

### Biological basis of deep learning networks

**Fig 5:**
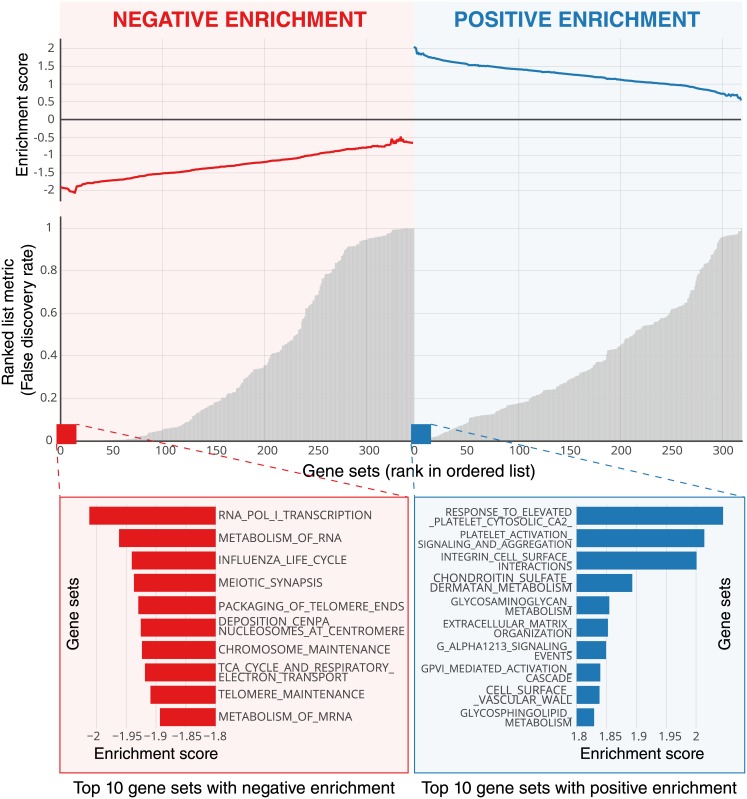
Global gene set expression patterns—Moffitt dataset. The deep learning network predictions on the surgery training dataset Moffitt were linked to global gene expression patterns using a pre-ranked gene set enrichment analysis (GSEA). Negative and positive enrichments are shown in red and blue, respectively. The top 10 enrichments in each category are highlighted. See S1 File for full ranking and associated enrichment scores.

We also explored the biological basis of the radiographic phenotypes quantified by the CNN through investigating imaging and gene expression assays in the surgery training dataset Moffitt (*n* = 200). We linked the CNN predictions to global gene expression patterns using a pre-ranked GSEA. Notably, the majority of the most significantly enriched pathways (FDR ≤ 10^−3^) are directly linked to cell cycle and transcriptional processes (Fig 5; S1 File). For example, meiotic synapsis, telomere packaging, and various cell cycle stages such as G1 and S were among the top associations. Notably, these enrichments were highly negative—thus suggesting that the network predictions show inverse correlation to a proliferating phenotype. These results were consistent when reproduced in the surgery tuning dataset MUMC (*n* = 90) (S5 Fig; S2 File), where cell cycle and proliferation pathways, as well as various transcriptional processes, were observed among the most significant associations.

## Discussion

In this study, we assessed the utility of deep learning networks in predicting 2-year overall survival of NSCLC patients from CT data. We trained a 3D CNN end to end on patients treated with radiotherapy, and employed a transfer learning approach for those treated with surgery. We demonstrated the CNN’s ability to significantly stratify patients into low and high mortality risk groups, as well as its stability in test–retest and inter-reader variability scenarios. In addition to benchmarking against feature engineering methods, we also highlighted regions with the largest contributions to the captured prognostic signatures, both within and beyond the tumor volume. Finally, our preliminary genomic association studies suggested correlations between deep learning features and cell cycle and transcriptional processes.

This effort builds upon a body of deep learning applications in medical imaging that has emerged since the unprecedented superior performance of CNNs in recent image classification competitions [55]. Few deep learning studies to date have explored prognostication, with most addressing other tasks including segmentation, detection, and malignancy classification [17]. While feature definition is automated in these deep learning approaches, radiomics has primarily relied on the extraction, selection, and subsequent classification of predefined features using other machine learning methods including shallow neural networks, random forests, and support vector machines among others [15]. These methods have found applications in the prognostication of nasopharyngeal carcinoma in MRI [56], pulmonary adenocarcinoma in CT [57], and early-stage NSCLC in positron emission tomography (PET)/CT [58] to name a few. Consequently, in this study, we benchmarked the deep learning networks against random forest models built on engineered features, with the performance of the random forest models being within previously observed ranges [10]. These models exhibited an inferior performance when compared to the deep learning networks, although this difference was only significant for surgery patients. These results may be due to the higher levels of abstraction inherent in deep learning features over their engineered counterparts. Additionally, and in terms of input formats, engineered features were extracted exclusively from within tumor annotations. Deep learning inputs, however, were comprised of 3D cubes allowing the network to consider tumor-surrounding tissue. This effect is magnified in the smaller tumors treated with surgery relative to their larger radiotherapy counterparts, potentially explaining the significance of the surgery results. Surgery patients are often excluded from engineered radiomics studies [59–61], where no prognostic signal has been detected, with the cited reasoning being the lack of rationale in predicting a tumor response based on its phenotype if it is resected. Our results hint at the potential utility of deep learning networks in stratifying this specific patient group.

We also explored models built on a set of clinical features, including age, sex, and TNM stage. These models performed poorly in both the radiotherapy and surgery datasets, potentially due to the limited features available and common to all 6 datasets. Imaging features commonly used in the clinic, namely tumor volume and maximum diameter, performed relatively well on the radiotherapy datasets, but rather poorly on the surgery datasets, as has previously been demonstrated [62]. Both models were outperformed by deep learning approaches, although the difference was only significant for the surgery datasets. Further studies are needed to investigate the prognostic relationship between these features and deep learning features for radiotherapy patients, especially given the well-established relationship between tumor volume and survival in this group [63]. These results also hint at the prognostic superiority of deep learning features for surgery patients.

Our efforts to identify salient regions within images through activation mapping hint at the significance of tumor-surrounding tissue in patient stratification. This aligns with efforts that showcase the prognostic value of tumor location [64] as well as the importance of understanding the interactions between tumors and their surroundings as a means for effective cancer prevention and care [65].

Finally, our preliminary genomic association study showcases correlations between the deep learning network predictions and cell cycling, transcriptional, and other DNA replication processes, such as DNA repair or damage response. This suggests that deep learning features may be driven by underlying molecular processes mostly related to proliferation of cells and hence progression of tumors. Moreover, nearly all significantly enriched biological processes had a negative enrichment score, indicating an inverse relationship to the survival predictions. This suggests that the gene expression present in cell proliferating pathways tends to be downregulated, with higher network scores indicating a higher survival probability. As associations between engineered imaging features and biological pathways have already been established [14,66], our study extends these associations to deep learning.

Strengths of this study include the relatively large—in cancer imaging terms—set of 1,194 NSCLC patients with training, tuning, and testing on independent datasets. The datasets were heterogeneous in terms of imaging acquisition parameters, clinical stage, and management, thus reflecting clinical reality. This suggests that deep learning methods may eventually be sufficiently robust and generalizable for practical application in clinical care. In addition to being a non-invasive and cost-effective routine medical test [67,68], CT imaging provides a relatively stable radiodensity metric standardized across equipment vendors and imaging protocols compared to other imaging modalities (e.g., MRI and PET). In comparison to engineered radiomic methods that require slice-by-slice tumor annotations—a time consuming and expensive process that is highly prone to inter-reader variability—our approach may yield higher throughput as it only requires a single-click seed point placement roughly within the center of the tumor volume. The 2-year survival endpoint utilized here is a relevant survival cutoff for NSCLC patients and one that has been previously used in prognostication efforts [69–71]. Our study hints at the utility of transfer learning within medical imaging and across treatment types, a finding that is also strengthened through benchmarking against end-to-end training of the surgery training dataset (S6 Fig).

Several limitations should also be noted. By design, the retrospective nature of this study hindered the ability to gauge how and where such a tool can potentially be integrated into the clinical workflow. Consequently, the prognostic knowledge distilled into the deep learning networks is based on earlier treatment options and protocols, and may not be adequately positioned to infer a prognostic signature for a patient treated with more modern means. The opaqueness of deep learning networks is another limitation. Feature definition, extraction, and selection in these methods—a major source of variability in engineered radiomics [15]—are all automated and occur implicitly. This comes at an expensive cost: interpretability. Consequently, these black-box-like networks are very difficult to debug, isolate the reason behind certain outcomes, and predict when and where failures will happen. Without a strong theoretical backing [72], deep learning features are nameless, and the imaging characteristics they measure are highly obscure. This ambiguity is in sharp contrast to the expert-based well-defined engineered features, and is often exacerbated in prognostication problems where the only means of validation is long-term mortality follow-up through prospective studies. Additionally, a better understanding of the network hyper-parameter space is needed, potentially provided by using multiple tuning datasets within the discovery phase and prior to the final test phase. Another limitation lies in the input data space. Despite the aforementioned dataset heterogeneity, CT stability, and test–retest and inter-reader variability studies performed herein, the networks’ sensitivity to other variations in clinical parameters and image acquisition parameters, including tube current, noise index levels, and reconstruction-specific parameters among others, has not been explored. Finally, as the survival times used in this study are overall as opposed to being cancer-specific, they may be influenced by external factors and introduce uncertainty into the problem.

Given the fixed input size of the deep learning networks used in this study, future research directions include exploring classification network architectures that accept inputs of simultaneous multi-scale resolutions [73] or variable sizes [74]—an approach common to fully convolutional networks used in image segmentation. Inputs of varying scales can potentially allow for combining the large tumors in radiotherapy patients with their relatively smaller counterparts in surgery patients into one prognostic network whilst maintaining robustness against such variation. In terms of interpretability, training neural networks with disentangled hidden layer representations is an active area of research [75]. While our activation mapping studies offer a qualitative measure of network attention, a more quantitative visualization and diagnosis of network representations is needed, especially with applications in the medical space. Additionally, a safeguard against neural networks’ blind spots is required in addressing our weak understanding of their susceptibility to adversarial attacks [76], and more specifically the sensitivity of medical images to certain reported counterintuitive properties of CNNs [77]. Finally, recent advances in imaging genomics [78] motivate further explorations beyond our preliminary GSEA study. When rigorously evaluated in future prospective studies, deep-learning-based prognostic signatures could highlight the specific biological states of tumorigenesis exhibited by a given patient, and thus enable more targeted therapy applications that exploit specific biological traits.

The development of prognostic biomarkers for NSCLC patients is an active area of research, where tumor staging information is augmented with radiographic, genetic, molecular, and protein-based evidence [79,80]. The lack of a truly prognostic clinical gold standard hinders the ability to accurately benchmark these biomarkers and further stresses the need for prospective validation. While TNM staging is often utilized in the clinic as the primary means for NSCLC prognostication and treatment selection, it is mainly intended as a discrete measure of tumor extent and a clinical communication tool, in addition to being simple and static by design. Conversely, quantitative imaging features inferred through deep learning are continuous and high-dimensional, and may be used to augment the higher level, coarser stratification provided by TNM staging. After considering the aforementioned limitations, a prognostic imaging tool may allow the transition to a finer classification enabling the identification of appropriate treatment plans on the individual patient level. One potential application for such transition may be in managing early-stage NSCLC patients, for whom surgery represents a therapeutic mainstay albeit having high recurrence risks [7]. Adjuvant chemotherapy is often administered as a means of reducing these risks [81,82]. While T and N stage are known to be associated with recurrence in these patients [83], we find that patients with similar clinical characteristics can exhibit wide variations in the incidence of recurrence [84] and survival [85]. A finer classification within the same stage may allow for identifying low and high mortality risk patients. Accordingly, low-risk patients may be spared the adverse physical and mental effects as well as associated costs of adjuvant chemotherapy, and, conversely, more stringent post-treatment surveillance of those at high risk may be planned. Additionally, a more detailed stratification could potentially inform surgical approaches and techniques, empower high-risk patients with the choice of adjuvant therapy modalities that best fit their desired lifestyles, and identify long-term beneficiaries from such therapy [86].

Deep learning algorithms that learn from experience offer access to unprecedented states of intelligence that, in some cases, match human intelligence. Beyond imaging, deep learning’s multimodal nature [87] promises the integration of multiple parallel streams of information spanning genomics, pathology, electronic health records, social media, and many other modalities into powerful integrated diagnostic systems [88]. Despite numerous roadblocks including the need for standardized data collection methods, evaluation criteria, prospective validation, and reporting protocols [89], the greatest anticipated clinical impact of these algorithms will be within precision medicine. This emerging approach allows for early diagnosis and customized patient-specific treatments, thus delivering the appropriate medical care to the right patient at the right time [90]. While medical imaging has always provided an individual assessment of ailments, AI algorithms based on imaging biomarkers promise to accurately stratify patients and enable new research avenues for personalized healthcare.

## Supporting information

S1 Checklist Strengthening the Reporting of Observational Studies in Epidemiology (STROBE) checklist. (DOCX)

S1 Fig Distribution of tumor bounding box dimensions in the radiotherapy training dataset HarvardRT. This distribution is based on ground truth tumor annotations and was used to determine and optimize the input size to the CNN. An input size of 50 × 50 × 50 mm was found to be optimum as it gave the best performance on the tuning dataset Radboud. Around 60% of all tumors are fully contained within this input cube, and the remaining tumors are cropped. While a larger input cube would allow for more context, it could potentially include non-predictive features such as bone tissue. Conversely, a smaller input cube would offer very little context, if any, and crop a large number of tumors.
(EPS)

S2 Fig Benchmarking deep learning networks against engineered and clinical models. This figure compares the prognostic performance of deep learning networks with random forest models. The benchmarking is based on predicting overall 2-year survival. The deep learning networks are used for reference with performance at AUC = 0.70 (95% CI 0.63–0.78, *p* = 1.13 × 10^−07^) and AUC = 0.71 (95% CI 0.60–0.82, *p* = 3.02 × 10^−04^) for the radiotherapy and surgery datasets, respectively. The random forest models are built on clinical parameters (age, sex, and TNM stage) and engineered features. This is in addition to tumor volume and maximum diameter models. Clinical data: Models built on clinical parameters returned AUC = 0.55 (95% CI 0.47–0.64, *p* = 0.21) and AUC = 0.58 (95% CI 0.39–0.77, *p* = 0.4) for the radiotherapy and surgery datasets, respectively. These models performed significantly worse than deep learning networks as demonstrated by permutation tests (*N* = 1,000) on the radiotherapy (*p* = 1 × 10^−06^) and surgery (*p* = 0.02) datasets. These results were confirmed with the meta *p*-value (*p* = 0.003). Engineered features: Models built on engineered features returned AUC = 0.66 (95% CI 0.58–0.75, *p* = 1.91 × 10^−04^) and AUC = 0.58 (95% CI 0.44–0.75, *p* = 0.275) for the radiotherapy and surgery datasets, respectively. As concluded using permutation tests (*N* = 1,000), these results were not significantly worse than those of the radiotherapy deep learning network (*p* = 0.132) but were significantly worse than those of the surgery deep learning network (*p* = 0.035). These results were confirmed with a meta *p*-value test (*p* = 0.06). Volume: Tumor volume returned AUC = 0.64 (95% CI 0.56–0.73, *p* = 6.18 × 10^−04^) and AUC = 0.51 (95% CI 0.37–0.66, *p* = 0.85) for the radiotherapy and surgery datasets, respectively. As demonstrated by permutation tests (*N* = 1,000), tumor volume did not perform significantly worse than deep learning networks on the radiotherapy dataset (*p* = 0.056) but performed significantly worse on the surgery dataset (*p* = 0.004). These results were confirmed with the meta *p*-value (*p* = 7.60 × 10^−05^). Maximum diameter: Maximum diameter returned AUC = 0.63 (95% CI 0.55–0.71, *p* = 2.15 × 10^−03^) and AUC = 0.50 (95% CI 0.35–0.66, *p* = 0.94) for the radiotherapy and surgery datasets, respectively. Maximum diameter did not perform significantly worse than deep learning networks as demonstrated by permutation tests (*N* = 1,000) on the radiotherapy dataset (*p* = 0.051) but performed significantly worse on the surgery dataset (*p* = 0.002). These results were confirmed with the meta *p*-value (*p* = 7.47 × 10^−05^).
(EPS)

S3 Fig Stability against inter-reader variations. To simulate human readers annotating tumor centers with some variability, we translated the input seed point in 3D space. (A) Translation distances along *X*, *Y*, and *Z* are drawn separately from a binomial distribution with probabilities based on a normal distribution (σ = 4). Translations are limited to a 30 × 30 × 30 mm cubic region surrounding the seed point. Here, we demonstrate this distribution over 2 axes only—actual translation occurred in 3 axes. The translation simulation is repeated 50 times. (B) Distribution of AUCs across the 50 runs.
(EPS)

S4 Fig Effects of tumor annotation information on prognostic power. The AUC plot illustrates the prognostic power of 3 different models as tested on the radiotherapy test dataset Maastro (*n* = 211). The first deep learning network, where the tumor volume is masked by giving regions beyond the tumor the value of air (−1,000 HU), is shown in green (AUC = 0.63). The random forest model based on engineered features, where tumor volume is completely masked, is shown in orange (AUC = 0.66). The second deep learning network, where the tumor volume is unmasked (Fig 3A), is shown in blue (AUC = 0.70).
(EPS)

S5 Fig Global gene set expression patterns—MUMC dataset. The deep learning network predictions on the surgery tuning dataset MUMC were linked to global gene expression patterns using a pre-ranked gene set enrichment analysis (GSEA). Negative and positive enrichments are shown in red and blue, respectively. The top 10 enrichments in each category are highlighted. See S2 File for full ranking and associated enrichment scores.
(EPS)

S6 Fig Benchmarking the effects of transfer learning—M-SPORE dataset. This plot illustrates the prognostic power of 3 different methodologies as tested on the surgery test dataset M-SPORE (*n* = 97). The first result (AUC = 0.71), shown in blue, represents the fine-tuned network with weights initialized from the radiotherapy network (Fig 3C). The second result (AUC = 0.56), shown in orange, represents a randomly initialized network trained end to end on the surgery training dataset Moffitt, utilizing the same hyper-parameters in the radiotherapy network (Methods). The third result (AUC = 0.48), shown in green, represents the radiotherapy network used as is (without fine-tuning any of its layers).
(EPS)

S1 File Gene set enrichment analysis (GSEA) for Moffitt dataset. (XLSX)

S2 File Gene set enrichment analysis (GSEA) for MUMC dataset. (XLSX)

S1 Table Dataset breakdown. Table showing the 7 datasets used in this study: 3 radiotherapy datasets, 3 surgery datasets, and 1 stability assessment dataset. Only patients with NSCLC and stages I through III were selected. For Kaplan–Meier curves and genomic association studies, all patients with survival follow-up were used. For deep learning and engineered feature studies, only patients with 2-year survival follow-up were used.
(EPS)

S2 Table Univariate Cox model results. Test results from univariate Cox models exploring the relationship between clinical factors and survival for both the radiotherapy and surgery patient groups. Deep learning was based on the median split from the respective tuning datasets. The same median split is used in the Kaplan–Meier curves (Fig 3B and 3D).
(EPS)

S1 Text Dataset information. (DOCX)

## Abbreviations

AI: artificial intelligence
AUC: area under the receiver operating characteristic curve
CNN: convolutional neural network
CT: computed tomography
FDR: false discovery rate
GSEA: gene set enrichment analysis
ICC: intraclass correlation coefficient
NSCLC: non-small-cell lung cancer
PET: positron emission tomography
TNM: tumor node metastasis
