# Supplementary material for: Deep learning for lung cancer prognostication: A retrospective multi-cohort radiomics study
Source: PLoS Med. 2018 Nov 30;15(11):e1002711. doi: 10.1371/journal.pmed.1002711 (PMC6269088; doi:10.1371/journal.pmed.1002711)
Supplement: S1 Text — (DOCX) [file pmed.1002711.s012.docx]

**S1 Text. Dataset information.
Harvard-RT** CTs were acquired for treatment planning purposes according to standardized scanning protocols using a GE “Lightspeed” CT scanner (GE Medical System, Milwaukee, WI, USA) with or without intravenous contrast. Tumor segmentation was manually performed on radiation therapy planning CTs using Eclipse software (Varian Medical System, Palo Alto, CA, USA). The primary tumor site was contoured using both soft tissue and lung windows by the treating radiation oncologists. Air, vessels, normal tissue or surrounding organs were subsequently excluded from these contours, and then individually verified by an expert radiation oncologist. Patients with prior treatment or induction chemotherapy prior image acquisition were excluded from the dataset. **Radboud**  All primary tumors and the mediastinal N2 disease were cytologically or histologically proven. All patients underwent diagnostic work-up, including contrast enhanced CT of the thorax and upper abdomen, whole body 18F-FDG-PET/CT, MRI of the brain, bronchoscopy with transbronchial needle aspiration (TBNA), and/or oesophageal ultrasound fine needle aspiration (EUS-FNA) and/or endobronchial ultrasound with TBNA (EBUS-TBNA) and mediastinoscopy in case of PET-positive, cytologically negative mediastinal lymph nodes. After work up, all patients were discussed in a thoracic oncology multidisciplinary board. Prior to radiotherapy a CT of the thorax was performed in radiotherapy position for radiotherapy planning.
 Patients in good general condition were treated with concurrent chemo radiotherapy, those with a contraindication for chemotherapy were treated by radiation alone, and all remaining patients were treated with a sequential chemotherapy and radiotherapy. The planned radiation dose to the primary tumor and metastatic mediastinal lymph nodes 28 using CRT until March 2008 and IMRT afterwards, was 66Gy in 33 fractions delivered five times per week. Chemotherapeutic agents in the sequential regimen typically consisted of three courses of gemcitabine (1250mg/m2; on day 1 and 8) and cisplatinum (80mg/m2; on day 1). The concurrent schedules varied between referring hospitals; in Radboud University Nijmegen Medical Centre it consisted of two courses of etoposide (100mg/m2; on day 1–3) and cisplatinum (50mg/m2; on day 1 and 8), in Canisius-Wilhelmina Hospital one course of gemcitabine/cisplatinum was administered prior to irradiation and two courses of etoposide/cisplatinum concurrently with radiation therapy. All research was carried out in compliance with the Helsinki Declaration and in accordance with Dutch law. The Institutional Review Board of the Radboud University Medical Center (RUMC) waved review due to the retrospective nature of this study. Follow-up was performed according to national guidelines. **Maastro** All patients received an FDG PET-CT scan for radiotherapy treatment planning, in radiotherapy position on a dedicated PET-CT simulator with both arms above the head. For the FDG PET-CT scans a Siemens Biograph (SOMATOM Sensation-16 with an ECAT ACCEL PET scanner) was used. An intravenous injection of (weight * 4 + 20) MBq FDG (Tyco Health Care, Amsterdam, The Netherlands) was followed by 10 ml physiologic saline. After a 45-min uptake period, during which the patient was encouraged to rest, PET and CT images were acquired. A spiral CT (3 mm slice thickness) with or without intravenous contrast was performed covering the complete thoracic region. Radiotherapy planning was performed on a XiO (Computerized Medical Systems, St Louis, Missouri) treatment planning system, based on a convolution algorithm using inhomogeneity corrections.
 Delineation based on fused PET-CT images was performed by the radiation oncologist by using a standard clinical delineation protocol. The protocol included fixed window level settings of both CT (lung W1700; L–300, mediastinum W600; L40) and PET scan (W30000; L15000) to be used for delineation. For all patients, a gross tumor volume (GTV) was defined based on FDG PET-CT data. All research was carried out in accordance with Dutch law. The Institutional Review Board of the Maastricht University Medical Center (MUMC+) waved review due to the retrospective nature of this study. This dataset is available online at <https://wiki.cancerimagingarchive.net/display/Public/NSCLC-Radiomics>.

**Moffitt**  This dataset consists of pretreatment diagnostic computed tomography (CT) scans, gene expression profiles, and clinical data for 262 patients treated in the Thoracic Oncology Program at the H. Lee Moffitt Cancer Center, Tampa, Florida, USA. We included patients with diagnosed primary tumors who underwent surgical resection and collected contrast-enhanced CT scans obtained within 60 days of the diagnosis between years 2006 and 2009. The majority of CT scans were recorded to be contrast-enhancing (89% of patients). Clinical data was available for 224 patients. Clinical outcomes investigated were overall survival (OS), pathologic TNM stage (combined T, N, and M stages, according to the latest version 7 of the IASLC guideline for lung cancer[[1]](https://paperpile.com/c/uOgYME/gKfDb)), and pathologic histology (grouped into adenocarcinoma, squamous carcinoma, and others). Clinical stage and histology were used when pathologic information was not available.

**MUMC** All primary tumors were cytologically or histologically proven. All patients underwent diagnostic work-up, including a CT scan of the thorax and upper abdomen with or without intravenous contrast and whole body 18F-FDG-PET/CT. After work up, all patients were discussed in a thoracic oncology multidisciplinary board. All patients were referred for surgical dissection. The CT scans had a slice thickness range between 1.5mm and 5mm. Primary tumors were segmented using the CT-based single-click ensemble segmentation algorithm[[2]](https://paperpile.com/c/uOgYME/2zVJ2). The study has been approved by the institutional review board of Maastricht University Medical Centre. All research was carried out in accordance with Dutch law. Informed consent was acquired for each patient included in this study.

**M-SPORE** The dataset was restricted to primary lung adenocarcinoma patients who had preoperative CT images available. For each patient, the datasets included de-identified diagnostic pre-treatment contrast-enhanced CT scans acquired between years 2006 and 2009 as well as clinical data including demographics, diagnosis, TNM stage, and patient survival. All CT scans were performed before surgery. The slice thickness varied between 1 and 5 mm (median: 5 mm). Tube voltage varied between 120 (118 patients), 130 (3 patients) and 140 (10 patients). Patient CT scans were segmented to identify tumors. Target lesions were volumetrically segmented using single click ensemble segmentation algorithm[[2,3]](https://paperpile.com/c/uOgYME/2zVJ2+w2xlv). The resident radiologist (over 2 years of experience) oversaw the segmentation boundaries on the CT slices.

**References**

1. [Mirsadraee S, Oswal D, Alizadeh Y, Caulo A, van Beek E Jr. The 7th lung cancer TNM classification and staging system: Review of the changes and implications. World J Radiol. 2012;4: 128–134.](http://paperpile.com/b/uOgYME/gKfDb)

2. [Velazquez ER, Aerts HJ, Gu Y, Goldgof DB, De Ruysscher D, Dekker A, et al. A semiautomatic CT-based ensemble segmentation of lung tumors: Comparison with oncologists’ delineations and with the surgical specimen. Radiother Oncol. Elsevier; 2012;105: 167–173.](http://paperpile.com/b/uOgYME/2zVJ2)

3. [Gu Y, Kumar V, Hall LO, Goldgof DB, Li C-Y, Korn R, et al. Automated Delineation of Lung Tumors from CT Images Using a Single Click Ensemble Segmentation Approach. Pattern Recognit. 2013;46: 692–702.](http://paperpile.com/b/uOgYME/w2xlv)
